# Vernal Catarrh

**Published:** 1915-10

**Authors:** G. D. Ayer

**Affiliations:** Atlanta, Ga.


### VERNAL CATARRH.

By Dr G. D. Ayer, M. D., Atlanta, Ga.

This short paper will be devoted to the probable cause and treatment of vernal catarrh. I shall make no attempt to describe the types and symptoms, as a complete description can be found in any text book on opthalmology.

As to the diagnosis, there should be no trouble, especially, no confusion with Trachoma, for when in doubt an examination by a competent bacteriologist will show eosinophiles in vernal catarrh, while in trachoma we may or may not find trachoma bodies but never eosinophiles.

I wish here to relate a case, with a report of examination of secretions from the eyes. Patient came to me with marked conjunctivitis and oedema of the lids to the extent that he was unable to open his eyes, complaining of intense itching and stinging. A diagnosis had been made by a specialist of orbital cellulitis, but an examination of the nose was negative. Several smears from the secretions of the eyes were made by Dr. Gerald Selby, with following report: Slide No. 1, Stained with Hastings stain, Slide No. 2, Stained with Loeffers Methyl Blue, Slide No. 1, Slide covered with pus cells, few epitheleal cells from conjunctiva, no bacteria, few lymphocytes found but large proportion of pus cells, 20 per cent. being eosinophillic.

Slide No. 2, found a large number of pus cells, a few epitheleal cells, and a few bodies one-quarter larger than polymorphoneuclear lucocytes, protoplasm clear, sky blue eccentrically placed neucleus about four microns in diameter, oval in shape, one or two similarly shaped and stained bodies found in each cell; these were one-half size of main body of neucleus. The body found resembles somewhat in size and appearance the ameba. Slide obtained six days later shows no eosinophiles and none of the above-described bodies.

As to the etiology of this disease, Weeks thinks it is due to a specific micro-organism not yet determined. Fuchs, who is our best authority, says the cause is unknown and adds that inasmuch as we are unable to cure the disease, the treatment must be limited to the amelioration of the subjective symptoms, that these patients get well after having the disease from two to four years and sometimes as long as twenty years without leaving any marked trace of the disease behind, so that an immunity, in some quickly, in others slowly, is evidently developed and in this respect it resembles some of the animal parasitic diseases, malaria, uncinariasis, etc.

I also wish to call your attention to the fact that these patients have no temperature and their blood examination always shows an increase in eosinophiles.

At the suggestion of Dr. Selby that these bodies might be animal parasites, I decided to try emetine hypodermatically and found after the first few injections that the eosinophiles and these bodies disappeared and marked improvement took place.

In the case reported here after the third injection and on the sixth day the secretions from the eyes showed an absence of both the eosinophiles and these bodies. I believe from the above findings we are warranted in making an investigation along the line that vernal catarrh is probably a parasitic disease, and my only excuse for calling your attention to this subject is to stimulate further investigation as to the etiology of a disease far more common than generally supposed.

I do not want to be understood as saying emetine cures vernal catarrh, as I realize that a number of cases are necessary to form any definite conclusion. I hope to also try a weak solution of emetine dropped into the eyes.

Since writing this paper I notice that Dr. H. F. Harris has found these bodies which he says resemble animal parasites, also degenerated cells.

501-3 Atlanta National Bank Bldg., Atlanta, Ga.

